# Plasticity of Visual Pathways and Function in the Developing Brain: Is the Pulvinar a Crucial Player?

**DOI:** 10.3389/fnsys.2017.00003

**Published:** 2017-02-08

**Authors:** James A. Bourne, Maria Concetta Morrone

**Affiliations:** ^1^Australian Regenerative Medicine Institute, Monash UniversityMelbourne, VIC, Australia; ^2^Department of Translational Research on New Technologies in Medicine and Surgery, University of Pisa and IRCCS Stella Maris FoundationPisa, Italy

**Keywords:** visual system, development, thalamus, hemianopia, blindsight, infants, pulvinar

## Abstract

The pulvinar is the largest of the thalamic nuclei in the primates, including humans. In the primates, two of the three major subdivisions, the lateral and inferior pulvinar, are heavily interconnected with a significant proportion of the visual association cortex. However, while we now have a better understanding of the bidirectional connectivity of these pulvinar subdivisions, its functions remain somewhat of an enigma. Over the past few years, researchers have started to tackle this problem by addressing it from the angle of development and visual cortical lesions. In this review, we will draw together literature from the realms of studies in nonhuman primates and humans that have informed much of the current understanding. This literature has been responsible for changing many long-held opinions on the development of the visual cortex and how the pulvinar interacts dynamically with cortices during early life to ensure rapid development and functional capacity Furthermore, there is evidence to suggest involvement of the pulvinar following lesions of the primary visual cortex (V1) and geniculostriate pathway in early life which have far better functional outcomes than identical lesions obtained in adulthood. Shedding new light on the pulvinar and its role following lesions of the visual brain has implications for our understanding of visual brain disorders and the potential for recovery.

## Reconsideration on the classic view of developing visual pathways

The infant human visual brain is immature at birth, and consequently vision during the first weeks of life is characterized by poor acuity, shape, and color perception. Gradually visual capacity matures over the first 8–9 months but some properties do not reach “adult-like” levels until later in life, around 10 years (Braddick and Atkinson, 2011). In contrast to this late and gradual development, motion perception is a property already present in the first few weeks of life. Infants are capable of discriminating motion-direction soon after birth (Ball and Tronick, 1971; Wattam-Bell, 1992; Náñez and Yonas, 1994), and although sensitivity to global-motion continues to mature slowly over the first 4–7 years in humans, and 2–3 years in macaque monkeys (Giaschi and Regan, 1997; Ellemberg et al., 2004; Kiorpes and Movshon, 2004; Hadad et al., 2011), it is already present at birth. Conventional opinion suggests that a newborn's interaction with the visual world initially draws on innate circuits in the superior colliculus, pre-tectal or thalamic nuclei—but not the lateral geniculate nucleus (LGN). One important piece of evidence that suggests a role of sub-cortical structures in mediating motion perception in human infants is based on the asymmetry of opto-kinetic eye movement reflexes (OKN) (Atkinson and Braddick, 1981), present only in the first few month of life. Brisk monocular OKN responses can be elicited in newborn infants but only by motion in the temporal-to-nasal direction. Early development of subcortical mechanisms - probably involving the superior colliculus, may mediate the eye-following response in this direction, while the directional sensitivity in the nasal-to-temporal direction, emerging later at about 10 weeks, may be mediated by cortical mechanisms (Braddick et al., 1992; Morrone et al., 1999). OKN measurements in young infants with a severe lesion of the visual cortex, or with only one hemisphere, have corroborated this view (Braddick et al., 1992; Morrone et al., 1999; Mason et al., 2003).

After approximately 2 months of age, the primary visual pathway through the LGN to the primary visual cortex (V1) becomes the dominant route for visual information (Braddick and Atkinson, 2011). This anatomical event appears related to function in the ordered appearance of orientation and spatial frequency selectivity, followed by direction selectivity, and finally stereoscopic depth perception. Similarly, it was also hypothesized that the visual cortex develops in a hierarchical fashion with higher-order areas developing later, driven by feed-forward projections from previously developed lower-order cortical areas (Guillery, 2005; Watanabe et al., 2010). The protracted development (after an early emergence) of global motion sensitivity was attributed to late maturation of higher-level motion areas, such as the middle temporal area (V5/MT+) (Braddick et al., 1992, 2003; Wattam-Bell, 1992; Mason et al., 2003; Guillery, 2005; Kiorpes and Movshon, 2014).

The most widely used technique to study the development of the temporal and spatial properties of the visual system in infancy has been by recording of visually evoked potentials (VEP); however, this technique does not allow researchers to isolate the various cortices responsible. To date there is scant evidence from awake infants showing how the various cortical areas of human visual cortex develop, although this knowledge is fundamental in evaluating normal function and determining clinical outcomes of neonates with cortical brain lesions. The difficulties in unveiling the functional maturation of newborn cortex arise from the few methods that can be used with success in infants in the first months of life. High density electroencephalography (EEG) and near-infrared spectroscopy (NIRS) are usually not particularly reproducible in the first few weeks of life.

Previous attempts to record the blood-oxygen-dependent level (BOLD) response by functional magnetic resonance imaging (fMRI) in infants, by stimulating the brain under sedation, have had disappointing results. In these cases, very little activity was recorded beyond V1 and an anomalous negative response was observed in synchrony with the stimulus presentation. Only recently has it been possible to record fMRI activity in 4–7 week infants who are conscious and look attentively at a visual stimulus (Biagi et al., 2015). The results are distinct and reveal that cortical processing of motion is more mature at this age than suggested by previous data. Overall, a well-established network of direction/coherence selectivity for visual areas, very similar to that of adults, have been measured: the full network of cortical motion areas—including V6, MT and associative vestibular-visual cortex—is active at this very early age with BOLD responses very similar to those observed in adults. These results suggest that direction selectivity develop at similar time in primary and associative cortices: an unexpected result given the accepted hypothesis of a slow, uniform and progressive maturation of the cortex.

These BOLD responses were selective to a subtle difference in properties between two well-balanced motion stimuli: one stimulus comprised dots moving randomly, the other dots moving coherently along circular and radial trajectories. To respond selectively to these two types of stimuli, many neural properties are necessary: one is direction selectivity, the other integration across space and along complex trajectories. The data showed that both these complex properties are functional at a very early developmental age. This holds not only for visual associative cortices, but also for multisensory area such as the posterior insula that receives and combines vestibular and visual inputs. The cortical mechanism responsible for perception of ego-motion seems to be fully functional, suggesting that infants may also have a sense of body position and the illusory perception of vection. In contrast, the response of V1 to motion is more immature compared with other areas. This last result suggests the need for reconsideration of the hierarchical model of development of visual cortex. Associative cortex, like MT+ and the posterior insular, are strongly innervated by thalamic input, in addition to those from the LGN conveyed through the optic radiation. In adult monkey and human, MT+ receive a strong input from cortico-cortical connections, but it is also recipient of direct input from the koniocellular layers of the LGN (Sincich et al., 2004; Bridge et al., 2008; Schmid et al., 2010) and from a direct retino-recipient region of the pulvinar, both which bypass V1. It has therefore been hypothesized that the early maturation of MT might result from an earlier maturation of this input from pulvinar (Warner et al., 2015).

There is anatomical evidence in the primates, including humans, that MT is an early maturing visual cortical area. Support for this comes from studies looking at the cellular maturation, myelination profile and behavior (Watson et al., 1993; Condé et al., 1996; Bourne and Rosa, 2006; Bedny et al., 2010; Mundinano et al., 2015). Furthermore, it has been suggested by Bourne and others that area MT has many properties of sensory primary areas and may play an important role in anchoring the development of the capacious visual cortex (Rosa, 2002; Bourne and Rosa, 2006; Mundinano et al., 2015). Both areas V1 and MT have a topography inherited from direct thalamic inputs. The purpose of an additional area to underpin development of visual associative areas might be functional important. It has been suggested that it may allow the development of complex capability, requiring additional areas and connectivity, without losing the capacity for rapid development of specific function, such as motion detection. However, a caveat for this is the need for direct retinothalamic innervation of area MT to underpin its early maturation. A neural substrate that provides a significant input to area MT in early life is the medial portion of the inferior pulvinar (PIm), which is recipient of retinal input and whose efferents directly innervate MT (Warner et al., 2015).

**Figure 1:**
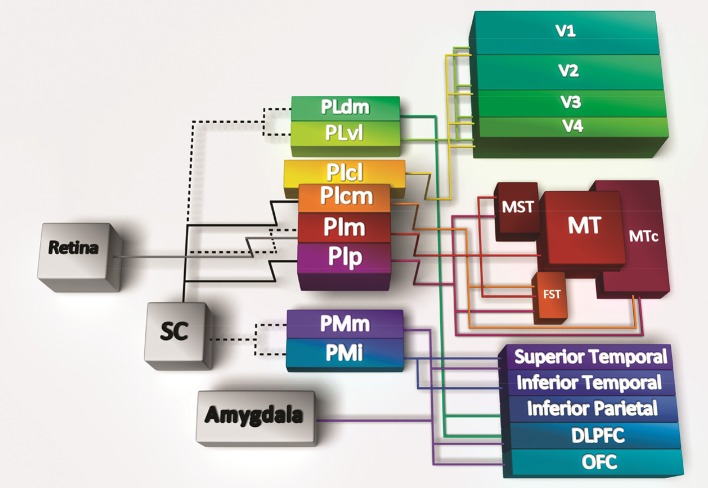
**Connectivity of the pulvinar subregions**. The pulvinar has significant reciprocal connectivity with the cortex, here summarized in cartoon form with lines depicting bidirectional connections except the connections from the retina and superior colliculus (SC), which are unidirectional. Hatched lines indicate reported connections that are controversial or have not been verified. Specific subdivisions within the inferior pulvinar (PI) and lateral pulvinar (PL) send and receive projections from both dorsal and ventral streams of the visual cortex. The medial subdivision of the inferior pulvinar (PIm) is recipient of input from the retina, and a disputed input from the superior colliuculus (SC; hatched line). The PIm in turn relays to the middle temporal (MT) area, the medial superior temporal area (MST), and the fundus of the superior temporal area (FST); all components of the dorsal stream. The central medial (cm) and posterior (p) subdivisions of the PI also connect with dorsal stream areas MST, FST, and the crescent of the middle temporal area (MTc). The central lateral subdivision of the PI (PIcl) and the ventrolateral (vl) subdivision of PL are heavily connected with the ventral stream [Reproduced with permission from Trends in Cognitive Sciences (Bridge et al., 2016)].

In both monkeys and humans, the pulvinar is located medial and dorsal to the LGN and is the largest of the thalamic structures. Furthermore, much less is known about the role of the pulvinar in comparison with other thalamic structures, although the past two decades have been instrumental in uncovering its anatomy in the anthropoid primates due to availability of new techniques to demarcate discrete regions and cell populations. The pulvinar comprises multiple subnuclei (Figure 1) that are arranged in a somewhat incongruous fashion without laminar structure. It is the inferior and lateral pulvinar that share significant connectivity with the visual cortex, while the medial pulvinar is more interconnected with the temporoparietal and frontal cortex. What is clear from the anatomy is that with the expansion of the visual cortex there has been a concomitant extension of the pulvinar subnuclei, suggesting a close relationship between the two. Unlike the LGN, the pulvinar is connected with a large portion of the extrastriate visual cortex, acting as a convergence point (Bridge et al., 2016). While human studies have generally been rather limited, a recent fMRI study demonstrated segregation into subdivisions mirroring the visual cortical dorsal/ventral distinction (Arcaro et al., 2015; Tamietto and Morrone, 2016), which was similar to that described for the nonhuman primates. A subset of nuclei in the inferior pulvinar connect predominantly to the dorsal stream, whereas more lateral nuclei connect to the ventral stream. The consistent homology between species has enabled a framework upon which to address specific hypotheses regarding the role of the pulvinar, especially in the developing brain.

## Development and maturation of visual pathways

**Figure 2:**
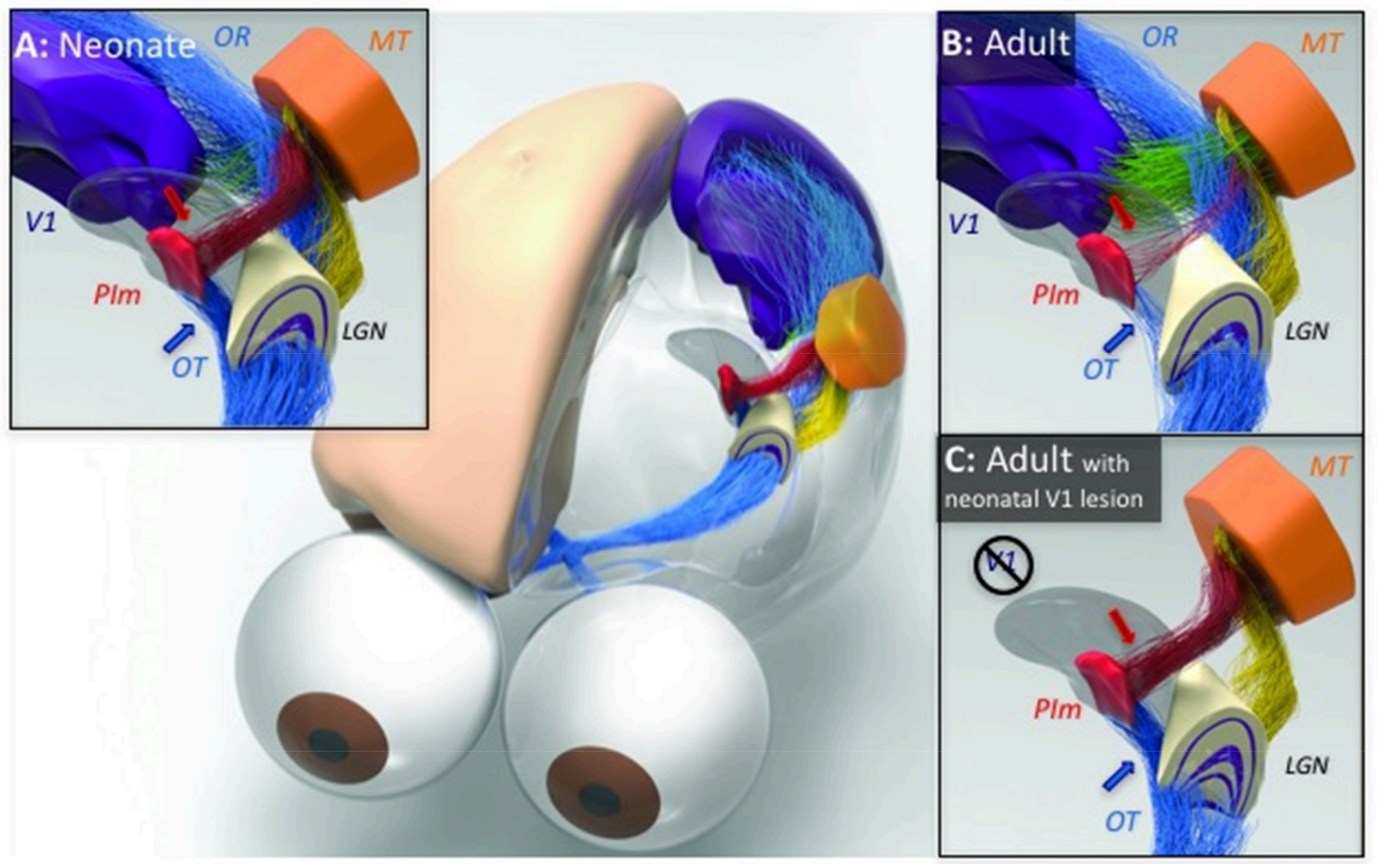
**Illustration of the developmental trajectory of the retino-pulvinar-MT pathway and the effects of early-life damage to V1, identified by neural tracing and imaging in the New World marmoset monkey**. **(A)** In the neonate, a prominent direct pathway (blue arrow) carries retinal information through the optic tract (OT) to the medial division of the inferior pulvinar (PIm), in addition to the lateral geniculate nucleus (LGN). A thalamocortical pathway from PIm (red arrow) is thought to pass this image information to cortical area MT, thus completing the early visual pathway to the extrastriate cortex. **(B)** During normal development, as the LGN pathway matures and begins to dominate visual input to the cortex through the optic radiations (OR), the early visual pathway through PIm regresses. **(C)** When animals develop in the context of an early life V1 lesion, this regression fails to occur. The LGN undergoes significant degeneration and both the afferent and efferent components of the PIm visual pathway remain intact. It may be for this reason that early life V1 lesions lead to a significant retention of vision. However, following a lesion of V1 in adulthood (not shown), the degeneration of the LGN is not accompanied by a strenghtening of the PIm-MT pathway, which has already regressed. [Reproduced with permission from Trends in Cognitive Sciences (Bridge et al., 2016)].

The two-stream hypothesis of visual processing in the cortex (Goodale and Milner, 1992) is a widely accepted model for both human and nonhuman primates. However, while there is a clear role during adulthood, few studies have investigated the development of the two streams and the functional relevance and consequences. Recently, studies in a New World simian, the marmoset monkey, with a comparable visual system to other primates (Mitchell and Leopold, 2015), have advanced a new standpoint on visual system development. These studies specifically revealed the transient nature of a pathway from the retina to the pulvinar without involvement of the superior colliculus (Warner et al., 2012), which is pruned during the postnatal period to a sparse projection by adulthood (Nakagawa and Tanaka, 1984; Cowey et al., 1994; O'Brien et al., 2001; Warner et al., 2010). Specifically, intraocular injection of anterograde tracer throughout the lifespan revealed a greater population of retinal afferent terminals in PIm in early life, especially in the first postnatal month of the marmoset, which represents an *in utero* period in other primates, including humans (O'Brien et al., 2001). Moreover, microscopic analysis revealed that the ganglion cells afferents terminated directly onto parvalbumin-positive relay neurons that directly project to MT (Figure 2, Warner et al., 2010), a cortical area heavily integrated and associated with the dorsal stream. The switch in dominance from the retinopulvinar–MT pathway to the LGN–V1 pathway is a major developmental milestone. As with the geniculostriate projections, the main pathway from V1 to MT is physically in place at this stage but likely yet to mature (Warner et al., 2010). After this time, MT receives most of its visual input from the visual cortices, and the pulvinar inputs decline in number. Among cortical areas, V1 sends prominent direct projections to MT. The increase in V1 input is concurrent with the decline of the PIm input, resulting in a change in the dominance of driving input to MT (Warner et al., 2012). Based on results from studies of other systems, this switch is likely to be accompanied by increased durability of the synaptic drive of V1 projection neurons in layers 2/3 (Stern et al., 2001), along with the development of perisomatic inhibition of projection neurons to the extrastriate cortex (Huang et al., 1999) and (Hensch et al., 1998), leading to a more honed visual topography (Mitchell and Leopold, 2015). In the adult, the retinal contribution to the pulvinar is strongly diminished (Figure 2B), with the primary driving input to virtually all of its subdivisions coming from the cortex.

These observations have led to the view that the visual pathway in which the PIm directly relays retinal information to MT is responsible for driving the early development and maturation of MT, as well as to support visually-guided behavior early in life. The connectivity between the retina, PIm and MT is present in greater quanta at birth (Figure 2A, blue and red arrows, respectively) but normally regresses in the first months of postnatal life (Figure 2B) in the marmoset monkey. Thus, once the retinopulvinar–MT pathway has served its role in shaping the development of the dorsal visual pathway, it becomes surpassed by the LGN–V1 pathway, whose detail vision and object specialization are critical for multiple aspects of primate visual cognition (Mitchell and Leopold, 2015). The monosynaptic retinopulvino-MT pulvinar is likely what directs the early maturation of the dorsal stream in comparison with the ventral stream, observed in multiple primate species, including humans, (Condé et al., 1996; Distler et al., 1996; Bourne and Rosa, 2006; Mundinano et al., 2015).

The data on human infants are consistent with a similar developmental trajectory observed in marmoset and point to the idea that during development vision can be influenced by alternative routes of sensory information. While it has been suggested that in the newborn it is the innate circuits through the superior colliculus that drives early visual processing, before the primary pathway through the LGN dominates, the pulvinar appears to be playing an integral role. The purpose for this likely arises from a teleological need to ensure a level of function to ensure survival in early life before the vast array of association areas becomes multiplexed together. Furthermore, it is apparent that this alternative route for visual information may play a crucial role during abnormal visual experience when V1 is damaged in early life.

### Abnormal pathway development: perinatal lesions of the visual cortex

Damage to V1 in the adult normally leads to the abolition of conscious vision (see later for a discussion of “blindsight”). However, studies have highlighted that primates, including humans, who receive damage to V1 in early life have a greater level of residual conscious vision. For example, infants who had experienced perinatal infarctions to V1 were much better in their visual performance than those who had acquired a similar injury during adolescence (Kiper et al., 2002; Knyazeva et al., 2002). Similarly, macaque monkeys who received a lesion to V1 at 2 months of age possessed more residual vision as adults than those with identical lesions obtained in adulthood (Moore et al., 1996). Therefore, in light of the observations outlined above, the most obvious candidate for the unusual conservation of visual perception following an early-life lesion of V1 might be the retinopulvinar–MT pathway which, while transient during normal development, may remain in place when the LGN pathway fails to evolve dominance.

This was highlighted in lesion studies in which the primary geniculostriate (LGN–V1) pathway was lesioned within the first couple of postnatal weeks, in the marmoset monkey, and the retinopulvinar–MT pathway persisted and remained robust into adulthood (Warner et al., 2015). Under such conditions, the retinopulvinar–MT pathway did not diminish after the first postnatal weeks, as was observed in the intact animals, but was sustained into adulthood (Figure 2C). This was true of both the retinal innervation of the PIm as well the relay to MT. Furthermore, in animals receiving adult V1 lesions, the retinopulvino-MT pathway remained comparable to the intact adult. These data suggest that in light of the apparent change in the dynamic pulvinar-associated developmental connectivity to MT, following removal of V1 and its efferents, this pathway must be subserving to function in the maturation of dorsal stream areas and associated behavior. However, to fully clarify the role the pulvinar pathway to MT plays in driving development of the dorsal stream, experimental lesions of PIm in early life are necessary.

**Figure 3:**
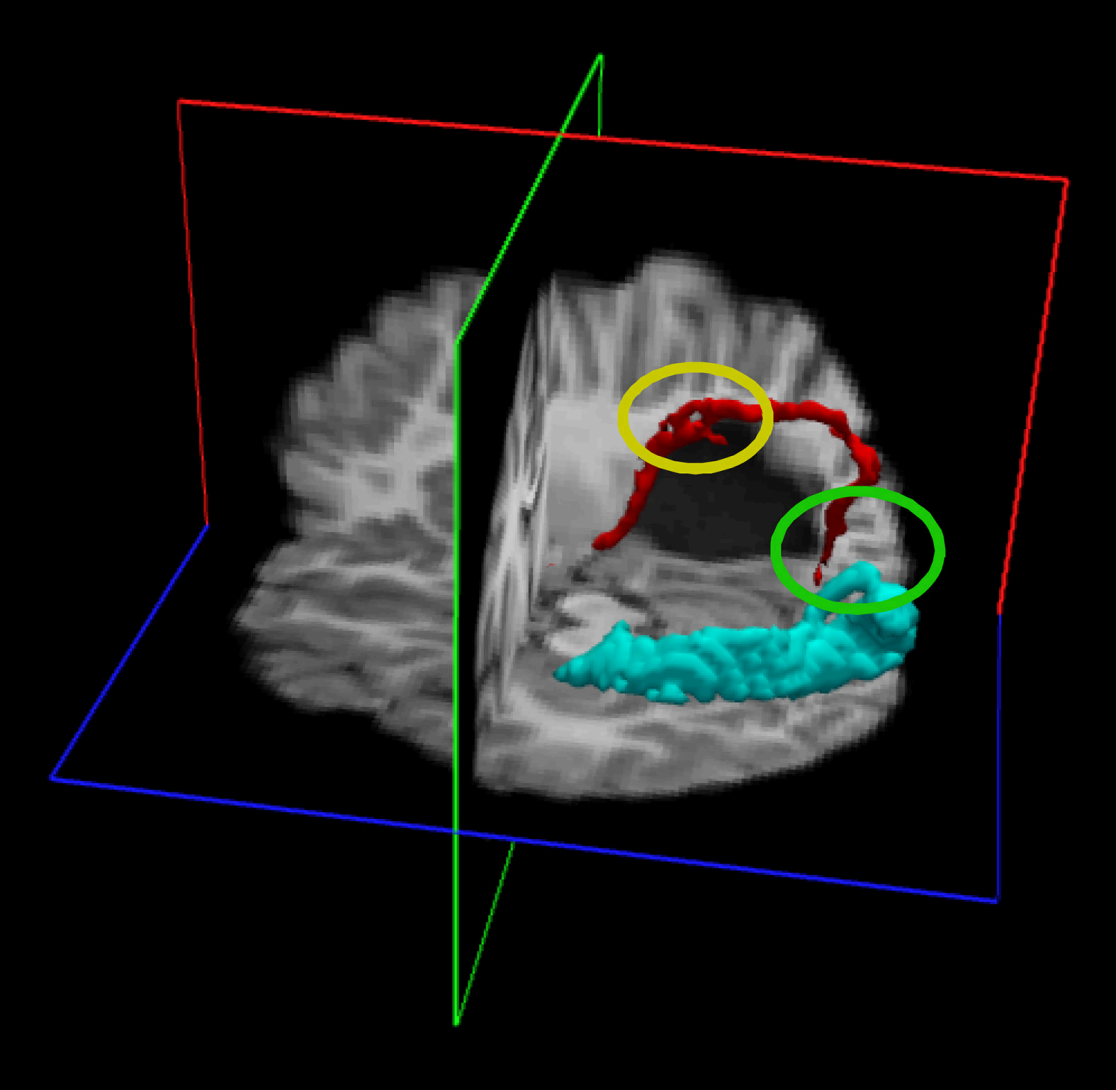
**Diffusion tensor imaging (DTI) of a patient with a left hemisphere lesion of the optic radiation caused by tumor compression during gestation (subsequent surgical removal of the tumor at 3 months of age)**. The subject has normal vision in the entire left visual field and in the central 30° of the right visual field. In the undamaged hemisphere, DTI shows the optic radiation follows a normal route (turquoise track). In the lesions hemisphere, DTI shows abnormal innervation of the calcarine cortex (red track); projections from the thalamus innervate first a region dorsal to the lesion that may correspond to the putative MT (highlighted by the yellow ellipse) and then the calcarine cortex (highlighted by the green ellipse). The two ROIs for the reconstruction of the tracks were positioned in an anatomical region encompassing the LGN and the Pulvinar and in the calcarine cortex. Red: the DTI fasciculus positioning the ROI in the LGN-Pulvinar and V1, with a waypoint in putative MT, of the lesioned hemisphere.

Recently the Pisa group studied an interesting patient who was born with a large gestational tumor of the left hemisphere, which presumably altered the visual pathways during *in utero* development (Aghakhanyan et al., 2004). The patient was operated at 3 months of age and the optical radiations were severely compromised by the surgery. Nevertheless, the child grew without any visual deficit and was referred only at age 7 years for visual screening for suspected dyslexia. The patient had only a marginal loss of form and contrast vision in the far periphery, despite the severe damage to the optical radiation. DTI result revealed the possible reason of the paradoxical contradiction between function and anatomy: in the lesioned hemisphere a strong connection was observed between an area that responded to motion (putative MT+) and a thalamic region close to LGN. The same connection was also present in the intact hemisphere, but it was very small (see Figure 3), while the optic radiations were normal. These results suggest that during development of the pathological brain, some abnormal thalamic projections can be formed. These can be normal projections that assume a different route because they are dislocated by the tumor, or may be totally new connections formed to partially compensate for the deficit. It is difficult to discern between these two alternatives. However, the strong analogy between the thalamic- MT connection in this patient with the persistence of retinopulvinar-MT projection in the marmoset with V1 lesions suggests that the thalamic-MT connection might arise from the retinopulvinar (it is very difficult with the current anatomical definition to discriminate between these two regions-of-interest in DTI studies). This might be transient in the normal human developing brain, but become stabilized given the loss of the optical radiation input.

### Blindsight in adult and congenital hemianopia: the role of the thalamic input to MT

Although the primary visual cortex (V1) is a fundamental station for visual information processing, subjects with lesions of V1 often have substantial spared visual function (Poppel et al., 1973; Weiskrantz et al., 1974; Barbur et al., 1980; Stoerig and Cowey, 1997; Radoeva et al., 2008). Residual vision for these patients is associated with a lack of consciousness, a condition termed *blindsight* (Weiskrantz et al., 1974). This is consistent with a key role of V1 in visual awareness. Subjects with blindsight are able to shift their gaze toward visual stimuli presented within the scotoma (Poppel et al., 1973; Weiskrantz et al., 1974), to point toward it (Danckert et al., 2003) and in many cases to discriminate the orientation (Morland et al., 1996), the direction of motion (Barbur et al., 1980), the spatial distribution (Sanders et al., 1974) and the wavelength (for a review see: Stoerig and Cowey, 1997) of the stimuli. Major anatomical and functional reorganization of neuronal circuitry that allows patients to “see without perceiving” have been observed. In the famous patient GY, an hemianopic subject whose right V1 was lesioned at the age of 8 years, Bridge et al. (2008) have shown, using DTI, abnormal contralateral connections between the right lateral geniculate nucleus (LGN) and the left MT+/V5, as well as callosal connections between the two MT+/V5 areas that are absent in controls. Both these aberrant connections bypass calcarine cortex. Abnormal contralateral projections from superior colliculus (SC) to associative and parietal visual areas, as well as V1 have also been observed (Leh et al., 2006). A more recent paper from Ajina et al. (2015) shows that human blindsight is probably mediated by an intact pathway between LGN and the middle-temporal visual area MT, but not from Pulvinar to MT+ (for review see Tamietto and Morrone, 2016). Ajina et al. (2015) subdivided a large group of patients with V1 damage into those with or without blindsight, according to a psychophysical test with moving visual stimuli. Diffusion-weighted magnetic resonance imaging (dw-MRI) and DTI were used to reconstruct white matter tracts that bypass V1. All patients with blindsight showed intact connections between LGN and extrastriate area MT. The converse was also true, as LGN–MT tracts were significantly impaired, or not detectable, in patients without blindsight. Alternative MT pathways that bypass V1 and originate from the ipsilateral superior colliculus and/or the pulvinar were also considered, but could not be consistently associated with the presence of blindsight. However, these other potential V1-independent pathways to MT, originating from the pulvinar and the superior colliculus, are difficult to dissect because these structures are so close together relative to the spatial resolution of tractography. It is also possible that the cortex does not need to be involved at all, at least in some forms of blindsight. For example, blindsight has been shown in patients with a hemispherectomy, where the entire cortex of one hemisphere has been removed (Leh et al., 2006).

Taken together, the data from several neuropsychological laboratories and evidence from monkey and cat lesion studies (Payne et al., 1996; Sorenson and Rodman, 1999; Lyon et al., 2010) indicate that SC and thalamus (LGN and pulvinar) may be key neuronal structures subserving blindsight (Tomaiuolo et al., 1997; Tamietto et al., 2010). As described earlier, thalamic projections can be relatively plastic during development.

**Figure 4:**
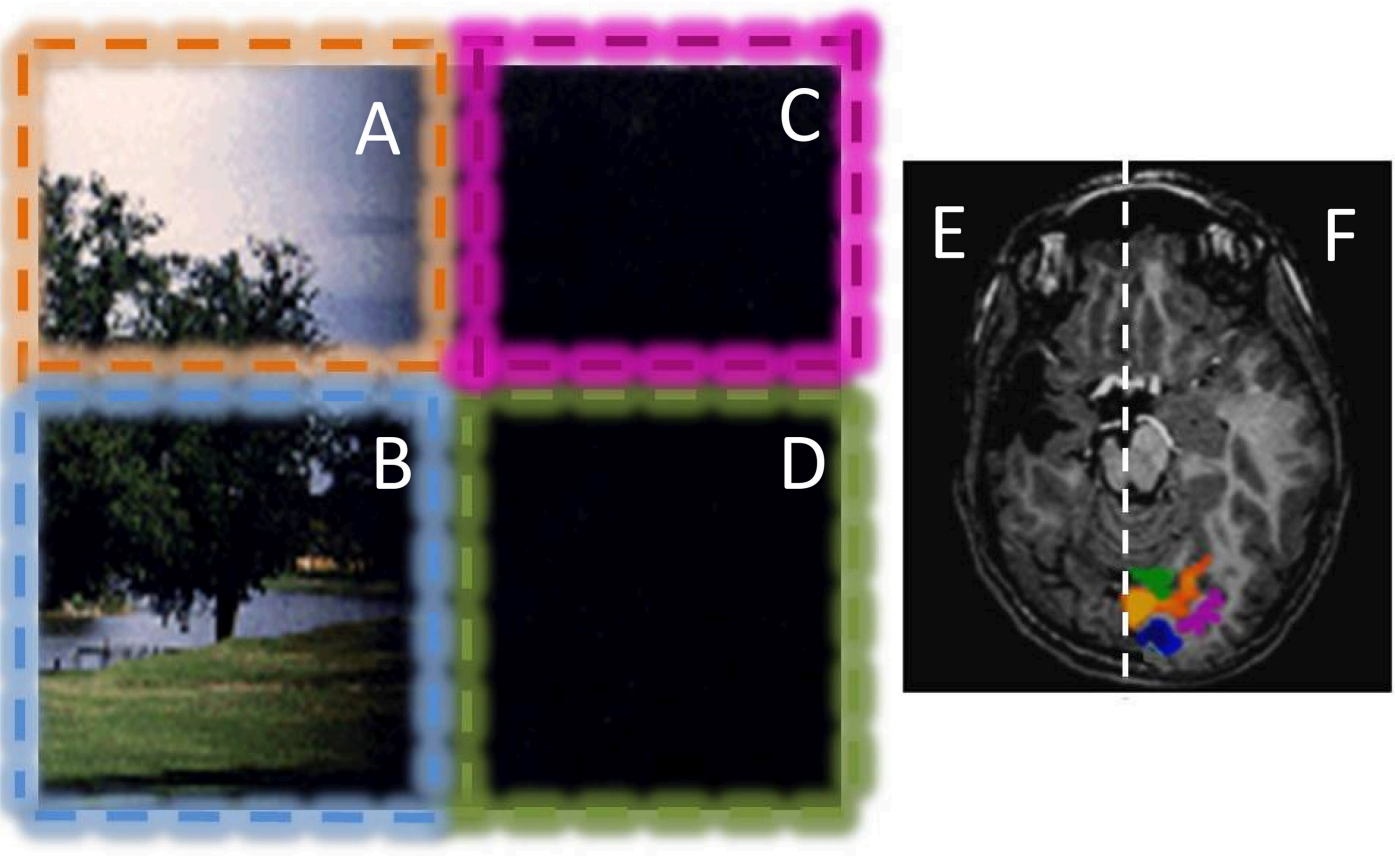
**Example of the visual field representation in the intact hemisphere of a hemianopic child with a congenital lesion**. The patient has dense hemianopia and no conscious vision for the right visual field. The vision of the patient is graphically represented in the left: Stimuli presented in the left upper (orange, **A**) and lower (blue, **B**) visual quadrants are always perceived; while stimuli presented in the right lower and upper visual quadrants (purple **C** and green **D**) are never consciously perceived. However, the patient can move efficiently in the environment, avoiding obstacles positioned in the blind visual field. He has a blindsight for orientation, alignment and motion. The optical radiation of the lesioned hemisphere are completely lesioned or degenerated and no BOLD activity was present in response to any visual stimuli in the lesioned hemisphere **(E)**. Stimulation of the left visual field elicits normal visual response with distinct representations for the upper (orange ROI in **F**) and lower visual field (blue ROI in **F**). Interestingly, stimulation of the blind hemifield elicits strong BOLD responses in the intact hemisphere (purple and green ROI in **F**), with an anomalous ipsilateral representation of the visual field. This type of ipsilateral representation of the visual field has been observed in many patients with congenital hemianopia, but not with acquired hemianopia, suggesting that it is mediated by a reorganization of the projections of pulvinar and/or SC to calcarine sulcus during development (see Tinelli et al., 2013).

One of the factors that makes blindsight more likely is the age at which the V1 lesion is acquired (Ptito and Leh, 2007), and patients with lesion during childhood are those that show a more profound neural reorganization (Leh et al., 2006; Bridge et al., 2008), like the extensively studied subject GY that became blindsigth at the age of 8 years old. In hemianopic patients, the probability that the scotoma shrinks during the years following brain injury (Teuber, 1975) correlates strongly with the age of the lesion in adolescents and young subjects (Teuber, 1975). Similarly, recovery of visual capabilities was greater in patients who underwent hemispherectomy at the age of 7 years, compared with cases where the surgery occurred later in life (Perenin, 1978; Kiper et al., 2002; Knyazeva et al., 2002). We compared congenital hemianopic patients with those who acquired similar optic radiation lesions during childhood to reveal the functional and anatomical reorganization potential of the human visual system in response to an early (perinatal) brain lesion (Tinelli et al., 2013); clear differences are apparent. First, all the congenital hemianopic children show a strong blindsight; they navigate in the room with nearly the same efficiency as normal children. When tested with forced choice on subtle visual properties, like spatial alignment of contrast-modulated targets, or on motion direction discrimination, they all performed significantly well. Given the profound lesion, the BOLD response in the lesioned hemisphere cortex did not respond to any visual stimulus, including all the dorsal area, and MT+ in particular. However, the visual cortex in the normal hemisphere did respond abnormally to both the contralateral and to the ipsilateral visual field. This effect was observed already at the level of V1 (see Figure 4). Given that these children had unilateral lesions of the optic radiation, and have large cortical and subcortical lesions, it is very difficult to imagine a crossed hemispheric pathway that can relay the signal from the ipsilateral visual field to the primary cortex. A possibility is again the strong pulvinar-MT projection, observed in the marmoset and in the patient GS described before. The ipsilateral visual signals could reach the pulvinar through several routes, including via superior colliculus. From pulvinar the signal would be first relayed to MT and then back to occipital cortex.

Consistent with the animal brain-lesion literature, the level of brain plasticity and reorganization potential at the time of lesion is an important property for the occurrence of blindsight. A massive rewiring of the visual system allows the extraordinary level of residual vision found in early lesioned animals and humans. The rewiring includes selective visual pathway reinforcement, neuronal degeneration and adjustment of neural activity (for a review see: Payne et al., 1996). The rewiring in our congenital patients may allow the robust V1 activation to ipsilateral stimulation in the scotoma. However, our patients do not have conscious perception of stimuli presented in the scotoma. This suggests that V1 is not sufficient for awareness, nicely complementing the argument (Ffytche and Zeki, 2011) that V1 is not necessary for awareness, implicating a variety of circuitries, including pulvinar circuitry, mediating consciousness.

## Author contributions

MM and JB wrote the review.

### Conflict of interest statement

The authors declare that the research was conducted in the absence of any commercial or financial relationships that could be construed as a potential conflict of interest.
